# Tensile and Compressive Mechanical Behaviour of Human Blood Clot Analogues

**DOI:** 10.1007/s10439-023-03181-6

**Published:** 2023-04-18

**Authors:** Rachel M. E. Cahalane, Judith J. de Vries, Moniek P. M. de Maat, Kim van Gaalen, Heleen M. van Beusekom, Aad van der Lugt, Behrooz Fereidoonnezhad, Ali C. Akyildiz, Frank J. H. Gijsen

**Affiliations:** 1 Department of Biomedical Engineering, Thoraxcenter, Erasmus MC, University Medical Center, Rotterdam, The Netherlands; 2 Department of Hematology, Erasmus MC, University Medical Center Rotterdam, Rotterdam, The Netherlands; 3 Experimental Cardiology, Erasmus MC, University Medical Center, Rotterdam, The Netherlands; 4 Department of Radiology and Nuclear Medicine, Erasmus MC, University Medical Center Rotterdam, Rotterdam, The Netherlands; 5 Department of Biomechanical Engineering, Delft University of Technology, Delft, The Netherlands

**Keywords:** Acute ischemic stroke, Mechanical thrombectomy, Thrombus, Material behaviour, Experimental testing, Histology, Composition

## Abstract

Endovascular thrombectomy procedures are significantly influenced by the mechanical response of thrombi to the multi-axial loading imposed during retrieval. Compression tests are commonly used to determine compressive *ex vivo* thrombus and clot analogue stiffness. However, there is a shortage of data in tension. This study compares the tensile and compressive response of clot analogues made from the blood of healthy human donors in a range of compositions. Citrated whole blood was collected from six healthy human donors. Contracted and non-contracted fibrin clots, whole blood clots and clots reconstructed with a range of red blood cell (RBC) volumetric concentrations (5–80%) were prepared under static conditions. Both uniaxial tension and unconfined compression tests were performed using custom-built setups. Approximately linear nominal stress–strain profiles were found under tension, while strong strain-stiffening profiles were observed under compression. Low- and high-strain stiffness values were acquired by applying a linear fit to the initial and final 10% of the nominal stress–strain curves. Tensile stiffness values were approximately 15 times higher than low-strain compressive stiffness and 40 times lower than high-strain compressive stiffness values. Tensile stiffness decreased with an increasing RBC volume in the blood mixture. In contrast, high-strain compressive stiffness values increased from 0 to 10%, followed by a decrease from 20 to 80% RBC volumes. Furthermore, inter-donor differences were observed with up to 50% variation in the stiffness of whole blood clot analogues prepared in the same manner between healthy human donors.

**Supplementary Information:**

The online version contains supplementary material available at 10.1007/s10439-023-03181-6.

## Introduction

Endovascular thrombectomy (EVT) is now the standard of care for acute ischemic stroke (AIS) patients with large vessel occlusions.^25^ Successful reperfusion is still not achieved in up to 20% of patients.^16^ EVT success is influenced by the mechanical response of the occluding thrombus to the multi-axial loading imposed during stent-retrieval or aspiration.^2, 14, 34^ Unconfined compression is commonly employed to determine compressive thrombus stiffness (resistance to deformation). However, the difficulties in thrombus material handling have led to a lack of stiffness data on thrombi in tension. Further, there are conflicting reports of linear and non-linear stress–strain responses in the limited tensile data available.^19^ Compressive test-derived behaviour is often used to model the complete clot EVT response,^20^ however, preliminary results suggest that ovine blood clot analogues exhibit compression-tension asymmetry.^9^

The mechanical properties of both retrieved thrombi and clot analogues made from the blood of different species have been examined to better understand the tissue response to EVT.^2^ The lack of access to EVT thrombi has led to the use of a wide range of species to produce clot analogues for mechanical testing.^2^ However, the mechanical response of human blood clot analogues could differ from the response of animal blood clot analogues. Moreover, the comparison of tensile and compressive loading responses of human blood clot analogues from the same donor is unknown. Thrombus composition impacts the outcome of EVT procedures,^4^ since fibrin-rich and RBC-rich thrombi have different mechanical behaviour under the same loading conditions.^2^ The physiological range of RBC content in thrombi retrieved from EVT procedures ranges from 0 to 90%.^1, 27^ The mechanical response of clot analogues should therefore be examined in a comparable range of compositions. Beyond RBC content, clot contraction also affects clot analogue mechanics.^13^ Platelets are the drivers of this clot contraction, mediating the compaction of the clot matrix and expulsion of serum.^3^ Correspondingly, the platelet content of *ex vivo* thrombi has also been associated with increased compressive stiffness.^1^

In this paper, we characterise the behaviour of human blood clot analogues in a range of compositions prepared in the same static manner for both tensile and compressive loading.

## Materials and Methods

### Sample Preparation

**Figure 1:**
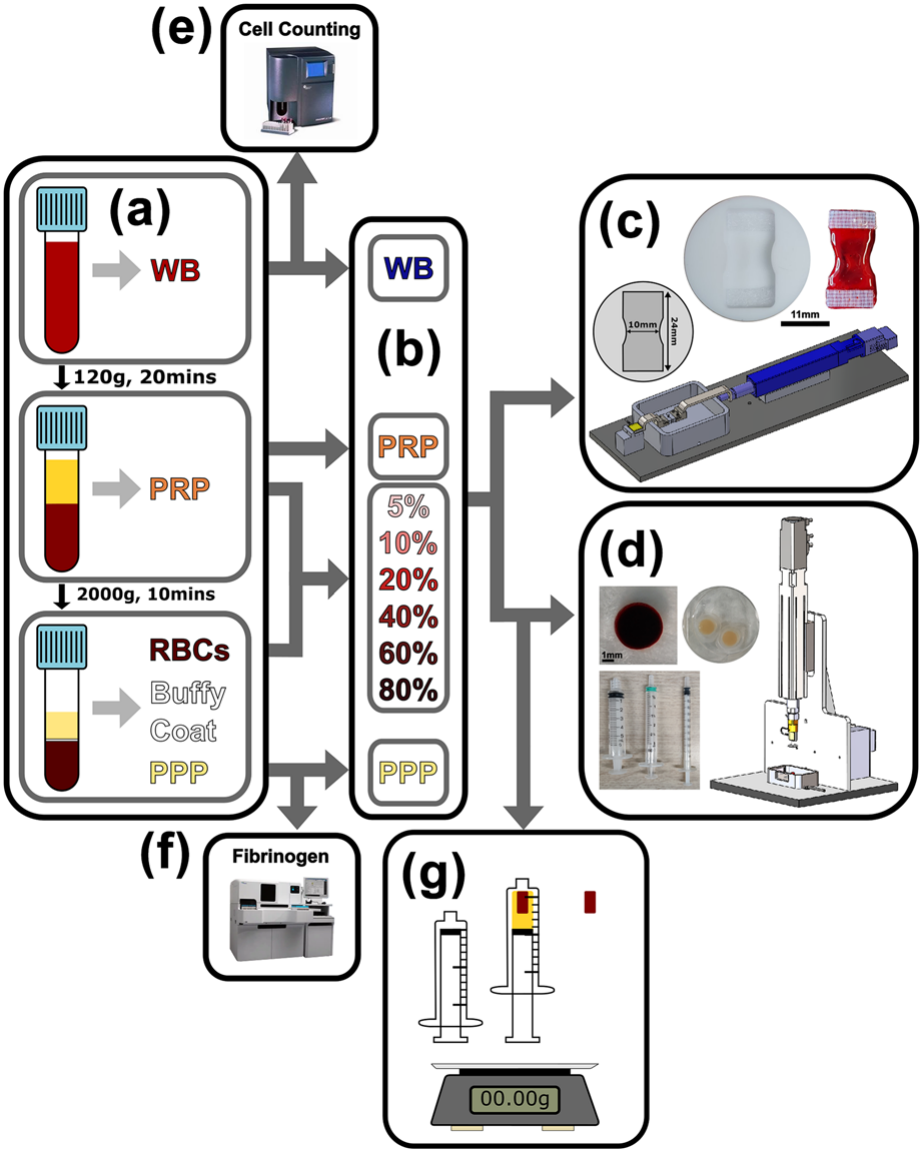
Overview of the experimental workflow. (a) Blood constituent acquisition. (b) Reconstructed clots and red blood cell (RBC) volumes. (c) Tensile mould, whole blood tensile sample and custom-built tensile setup. (d) Compression syringes, whole blood compression sample, platelet-poor plasma samples in Eppendorf lid and custom-built compression setup. (e) Whole blood cell counting. (f) Fibrinogen level quantification. (g) Clot contraction weight measurements: empty syringe, full syringe and clot.

Following a protocol approved by the Medical Ethical Committee at Erasmus MC (NL80622.078.22), blood was drawn by venepuncture from six healthy donors with no known haemostatic conditions and anticoagulated with 0.105 M buffered trisodium citrate. The blood was centrifuged at 120 *g* for 20 min at room temperature to obtain platelet-rich plasma (PRP) and then at 2000 *g* for 10 min to obtain platelet-poor plasma (PPP). The buffy coat layer was discarded, leaving the red blood cells (RBCs) (Fig. 1a). PRP, PPP and Whole Blood (WB) clots were prepared. To construct samples with a representative range of RBC concentrations, as observed with *ex vivo* thrombi,^1, 27^ the following volumetric percentages of RBCs were reconstituted with PRP: 5, 10, 20, 40, 60 and 80% (Fig. 1b). Coagulation was induced by adding CaCl_2_ (C5670, Sigma-Aldrich) and thrombin (T7009, Sigma-Aldrich) to final concentrations of 17 mM and 1U/mL in every sample, respectively. Clot analogues for tensile experiments were formed by adding the blood mixture to custom tensile moulds lined with Velcro tabs, similar to,^28^ and covered in mineral oil to prevent the formation of a fibrin biofilm^22^ (Fig. 1c). Clot analogues for compression experiments were formed by adding the blood mixture to either 5 mL (PRP and 5% RBC), 3 mL (10–60% RBC), 1 mL (80% RBC) syringes or Eppendorf lids (PPP) (Fig. 1d and S1). The blood volumes and syringes were chosen based on the degree of clot contraction to produce samples with a suitable cross-sectional area (CSA) for compression testing (Feret’s diameter at least 1.5 times the height). All clots were prepared at room temperature within 2 h of the blood draw and were contracted overnight at 37 °C in either a water bath (syringes standing vertically) or incubator (tensile moulds and Eppendorf lids in a six-well plate) (Fig.S1).

### Uniaxial Tension

Tensile experiments were conducted using a custom-built set-up (Fig. 1c). The samples were placed between a set of clamps and a pre-stretch was applied to remove the slack (Fig. S2E). The water bath was filled with a physiological medium at 37 °C. Samples were allowed to equilibrate for at least 5 min. An ultrasound scan was conducted using a Vevo 3100 Imaging System with an MX550S transducer (FUJIFILM VisualSonics, Canada). Samples were pre-conditioned using 10 cycles to 10% strain^29^ and then stretched until failure at a strain rate of 5%/s. It was not possible to perform tensile testing on the 80% RBC samples, as they were observed to be extremely fragile (Fig.S2F). Therefore, one sample of PPP, PRP, 5, 10, 20, 40 and 60% RBC volumes were prepared for each donor. To examine the reproducibility of the test method, two WB samples were prepared for each donor.

### Unconfined Compression

Compression experiments were conducted using a custom-built setup, previously described^1^ (Fig. 1d). Blood clots were cut to a height of 2 mm and photographed while submerged in a physiological medium. The samples were then transferred to a water bath containing a physiological medium at 37 °C. Samples were allowed to equilibrate for at least 5 min. Similar to the tensile experiments, samples were pre-conditioned using 10 cycles to 10% strain^29^ and then compressed to 80% strain at a rate of 5%/s. One sample of PPP, PRP, 5, 10, 20, 40, 60 and 80% RBC volumes were prepared for each donor. To examine the reproducibility of the test method, two WB samples were prepared and tested in the compression experiment where possible. Otherwise one WB sample was prepared and tested.

To conduct a preliminary examination of the intra-donor variation, a second blood draw was taken from donors 1, 3 and 4 at a later time point (t2) to make an additional set of samples for compression testing (n = 1 for each donor), scanning electron microscopy (SEM) and histology analysis (see Sect. "Scanning Electron Microscopy" and "Histology"). A complete overview of the samples prepared and tested is presented in Table S1.

### Mechanical Data Analysis

Cross-sectional measurements were acquired for the samples in the tensile experiments by taking the average of 3 measurements at a proximal, middle and distal location from the length of the ultrasound scan (Fig.S3A and B). Cross-sectional measurements were acquired for the samples in the compression experiments by taking 1 measurement of the cross-sectional area from the photographs (Fig.S3C and D). All measurements were performed in ImageJ. For both tensile and compression data, low- and high-strain stiffness values were acquired by applying a linear fit to the initial and final 10% of the nominal stress–strain curves, respectively.

### Hemostasis

Donor age and sex was recorded. RBCs, platelets and white blood cells were counted with a Coulter Counter (COULTER® AC•T diff™ Analyzer, Beckman Coulter, CA) (Fig. 1e). Fibrinogen levels were measured using the Clauss assay (Thrombin Reagent, Siemens Healthineers, Erlangen, Germany) on the Sysmex CS5100 coagulation analyzer (Siemens Healthineers, Diagnostics B.V., Newark, DE, USA) (Fig. 1f). Since contraction has been shown to alter clot analogue mechanics, the degree of clot contraction was assessed gravimetrically as the percentage weight of the expelled serum compared with the weight of the total blood mixture (Fig. 1g).^13^

### Scanning Electron Microscopy

Considering that the fibrin network structure is altered by the presence of RBCs,^11^ a part of the clot from the additional set of compression samples described in Sect. "Unconfined Compression" was prepared for SEM. These experiments were necessary to inspect the effect of RBC volumes on blood clot analogue structure. Samples were cut into two parts for SEM analysis and histology analysis. The part of the clot for SEM analysis was fixed in 4% buffered paraformaldehyde and 2% glutaraldehyde in 0.073 M sodium phosphate buffer pH 7.3 for 48 h at room temperature. Samples were then dehydrated in increasing grades of ethanol concentration, frozen in liquid nitrogen and fractured to expose the internal surface. The samples were then chemically dried with hexamethyldisilazane, mounted and sputter-coated with gold. SEM analysis was conducted using a JEOL JSM-6060LV microscope with an accelerating voltage of 5 kV.

### Histology

Considering that the volumes of RBCs used in the blood mixture do not produce blood clot analogues with proportionate RBC content,^8^ the second part of the clot as described in Sect. "Scanning Electron Microscopy" was prepared for histological analysis. The samples were fixed in 4% buffered formaldehyde for 48 h at room temperature, embedded in paraffin and cut into 5 *μ*m sections. Sections were stained using Martius Scarlet Blue (MSB) and CD42b. Quantitative analysis of components was performed using Orbit Image Analysis software.

### Statistical Analysis

Shapiro–Wilk analysis was performed to assess the distribution of continuous data. All data determined to be normally distributed were summarised as mean ± SD and presented in bar charts. Comparisons between RBC volumes were conducted using one-way ANOVA with Tukey posthoc analysis. Comparisons between contracted (PRP) and non-contracted (PPP) fibrin clots were conducted using independent t-tests. The association between donor demographics or blood composition measurements and whole blood clot mechanics as well as the degree of RBC volume and degree of clot contraction were examined using Pearson’s product-moment correlation coefficients (r_p_). The coefficient of variance (CoV) was used to quantify the amount of variation. All statistical analyses were conducted using SPSS v27. A *p*-value < 0.05 was deemed statistically significant.

## Results

### Donor Details

A total of 6 donors were included in this study (3 males, 3 females, age range 24–56). Donor demographics, whole blood cell counts and fibrinogen levels are presented in Table 1, which are all within the normal range for healthy human adults.

**Table 1:** Donor demographics, whole blood cell counts and fibrinogen levels.

Donor ID	Sex	Age (years)	Hematocrit (fraction)	Platelets (× 10^3^ cells/*μ*L)	White Blood Cells (× 10^3^ cells/*μ*L)	Fibrinogen (g/L)
1	Female	36	0.40	262	6.00	2.60
2	Male	24	0.44	185	6.47	2.30
3	Female	24	0.39	192	7.10	2.70
4	Male	54	0.42	244	5.77	3.30
5	Male	39	0.52	160	4.77	2.80
6	Female	56	0.45	242	7.10	4.20

### Nominal Tensile and Compressive Stress–Strain Profiles

**Figure 2:**
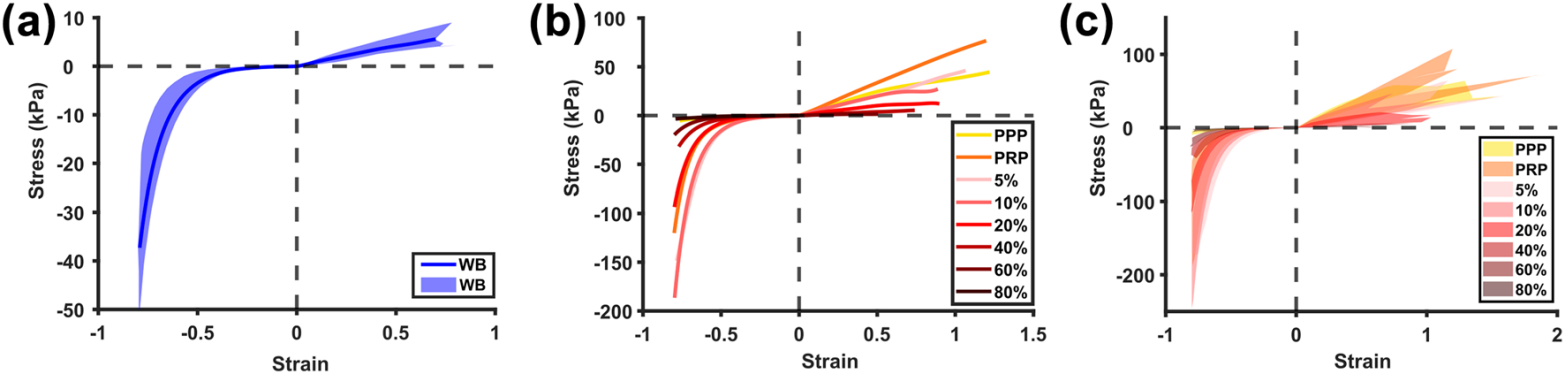
Nominal compressive and tensile stress–strain behaviour. (a) Whole blood midline and range shaded in blue. (b) Midlines per red blood cell (RBC) volume in the blood mixture. (b) Shaded range per RBC volume in the blood mixture.

A total of 114 samples were prepared and mechanically tested. Of these, 7 samples were excluded from the analysis for reaching the maximum value of the load cell (n = 2) and for slipping during testing (n = 5) (Table S1). Figure 2 presents the nominal stress–strain curves for each composition in compression and tension. The samples demonstrate a highly strain-stiffening compressive response but a generally linear tensile response. The same asymmetric profile is observed for all compositions. Variation is observed in Fig. 2c as the stress–strain range in response is overlapping for clots of different compositions from different donors. Individual stress–strain curves for each RBC concentration are presented in Figure S4.

### Tensile and Compression Stiffness

**Figure 3:**
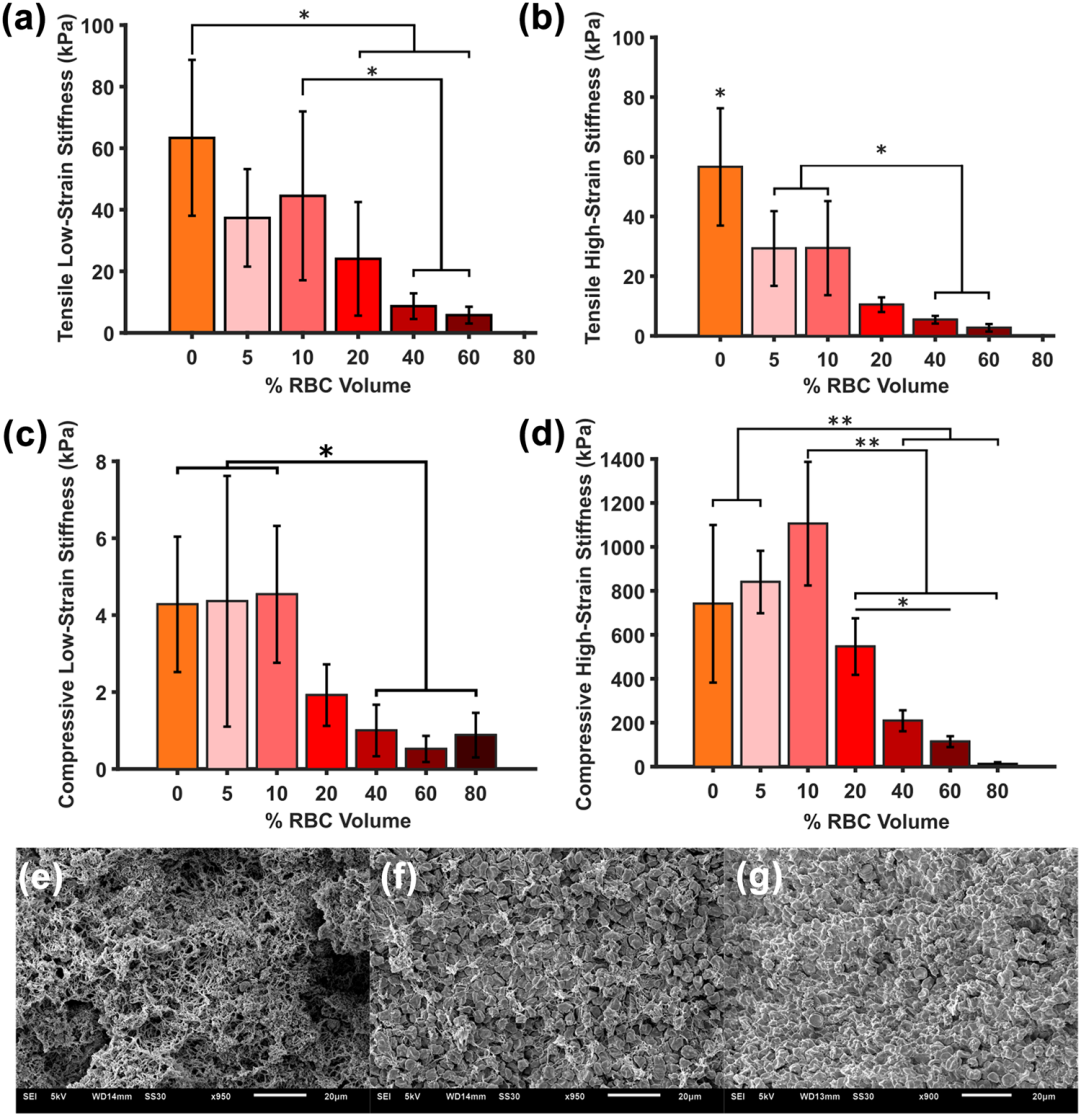
Influence of blood clot analogue red blood cell (RBC) volumes on mechanical stiffness and microstructure. (a) Tensile low-strain stiffness. (b) Tensile high-strain stiffness. (c) Compression low-strain stiffness. (d) Compression high-strain stiffness. (e) Platelet-rich plasma clot microstructure. (f) 10% and (g) 80% RBC volume clot microstructure. Statistical significance: *p ≤ 0.05 **p ≤ 0.005.

Figure 3a-d presents the tensile and compressive low- and high-strain stiffnesses per clot type. Tensile low and high-strain stiffnesses are not significantly different to each other (Fig. 3a, b). In contrast, compressive high-strain stiffness values are significantly higher than low-strain stiffness values for all compositions (*p* < 0.05). Tensile stiffness values are approximately 15 times higher than low-strain compressive stiffness and 40 times lower than high-strain compressive stiffness values. For the 80% RBC samples in compression, 4 out of 6 fractured prior to 80% strain.

We found non-linear associations between the RBC volume of the blood mixture and the mechanical properties. Tensile low-strain stiffness values appear to decrease from 0 to 5% RBC volume (63–37 kPa), followed by a slight increase at 10% RBC volume (45 kPa), and subsequently decrease for 20 to 60% RBC volumes (24–6 kPa) (Fig. 3a). For high-strain tension we observed a similar decrease from 0 to 5 and 10% RBC volumes (57–29 kPa), and further decreases in stiffness from 20 to 60% RBC volume (10–3 kPa) (Fig. 3b). Compression low-strain stiffness values appear relatively constant for 0 to 10% RBC volumes (4 kPa), followed by a decrease for 20–60% RBC volumes (2–0.50 kPa) with a slight increase at 80% RBC volume (1 kPa) (Fig. 3c). Compressive high-strain stiffness values exhibited the most apparent trend with RBC volume (Fig. 3d), increasing from 0% (741 kPa) up to the addition of 10% RBC volume (1106 kPa), and subsequently decrease from 20 to 80% RBC volume (546–12 kPa) (Fig. 3d). The stiffness at 10% RBC volume is almost statistically significantly higher than that at 0% RBC volume (*p* = 0.066). This implies that 10% RBC volume represents an inflection point at which the high-strain compressive stiffness ceases to increase and starts to decrease with increasing RBC volumes in the blood mixture.

From the SEM images, it was observed that PRP clots contain pores in the network with the absence of RBCs (Fig. 3e). RBCs are tightly packed in the network for a 10% RBC sample with polyhedrocytes present (Fig. 3f). However, for an 80% RBC volume sample, the network contains less fibrin and more biconcave RBCs, indicative of the absence of clot contraction forces (Fig. 3g). Representative SEM images for each RBC volume clot type are presented in Figure S5.

**Figure 4:**
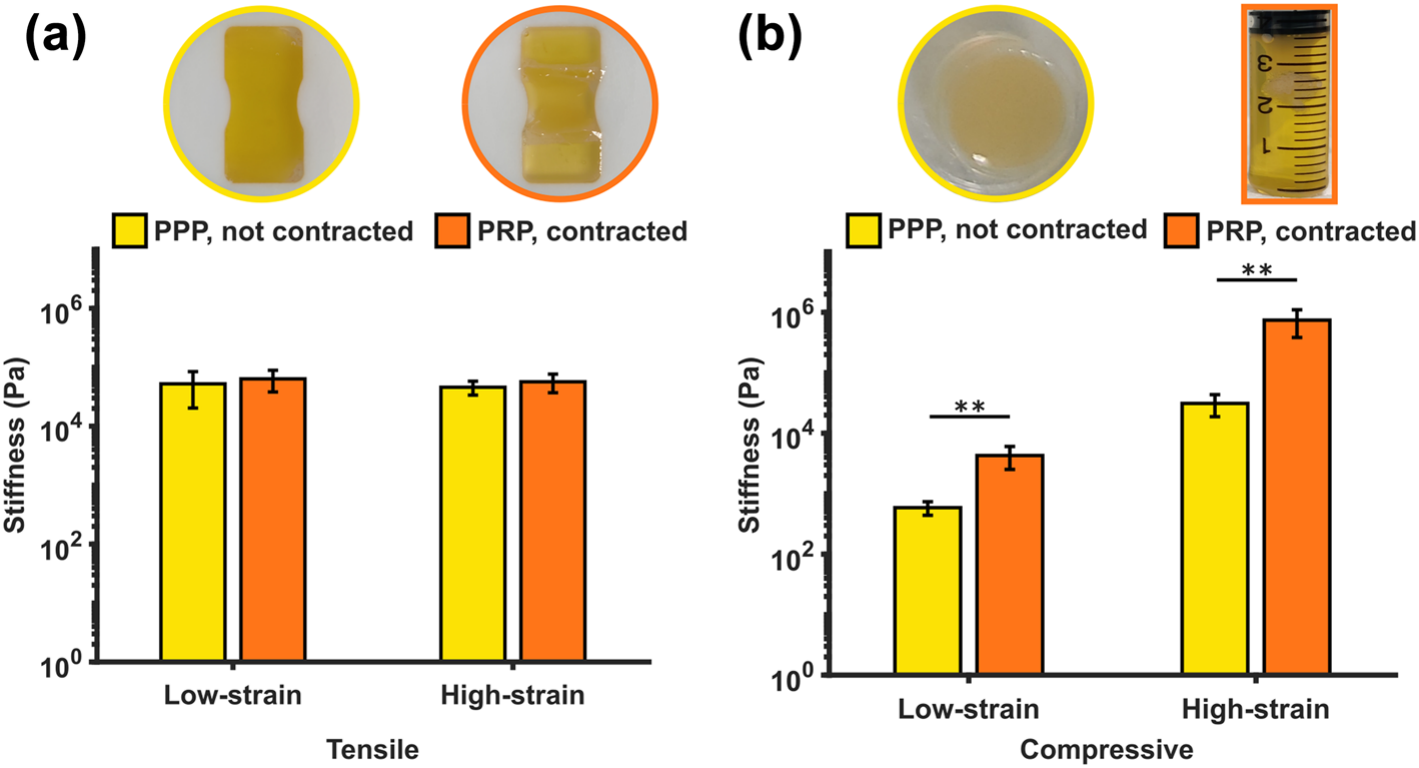
Influence of clot contraction on mechanical stiffness. Platelet-poor plasma (PPP) samples are not contracted while platelet-rich plasma (PRP) samples are contracted due to the presence of platelets. (a) Tensile stiffness. (b) Compressive stiffness. Statistical significance: **p ≤ 0.005.

A comparison of PPP and PRP fibrin clot stiffness is presented in Fig. 4 (note a logarithmic scale is used). PPP clots represent non-contracted fibrin clots while PRP clots represent a contracted phenotype. Remarkably, there were no significant differences in the stiffness of PPP and PRP clots at low- or high-tensile strains (Fig. 4a). As expected, PRP clots were stiffer than PPP clots in compression at both low (*p* = 0.004) and high strains (*p* = 0.005) (Fig. 4b).

### Inter- and Intra-Donor Variation

As demonstrated in Fig. 2c there are variations in the behaviour of comparable clots from different donors. To further quantify inter-donor variations, we examined the WB clot mechanics. High-strain compressive stiffness exhibited the lowest CoV (30%) (range 122–312 kPa), followed by low-strain compressive stiffness (47%) (0.14–1.29 kPa), low-strain tensile stiffness (48%) (3.59–14.67 kPa) and high-strain tensile stiffness (49%) (3.20–9.55 kPa). CoV for all other compositions is presented in Table 2 (44 ± 18%, range 17–77%). No other recorded parameters (blood cell counts, fibrinogen level or degree of contraction) exhibited CoV as high as the mechanical data (9–23%) (Table S2). Pearson’s correlation revealed trends towards significant correlations between platelet cell counts (*r*_p_ = 0.74, *p* = 0.09) and levels of fibrinogen (*r*_p_ = 0.71, *p* = 0.12) with high-strain compressive stiffness (Table S3).

**Table 2:** Coefficients of variance (%) for the stiffness results between donors for all compositions in tension and compression.

		Platelet-Poor Plasma	Platelet-Rich Plasma	5%	10%	20%	40%	60%	80%
Tensile	Low-strain	61	40	42	62	77	48	47	N/A
	High-strain	27	35	43	54	24	24	47	N/A
Compressive	Low-strain	25	41	75	39	42	67	66	66
	High-strain	40	48	17	25	24	23	22	69

To explore intra-donor variation in clot mechanics, a second set of WB clots for compression testing were prepared from a second blood draw for 3 of the donors (t2). The CoV for low- and high-strain compressive stiffness decreased to 28 and 26%, respectively. To confirm that the observed variation was not a result of experimental variation, an additional WB sample was analysed for 4 donors in tension and 3 donors in compression (t1). The CoV for these samples ranged from 7 to 25% (Table 3), which is within the limits of the other recorded parameters and lower than the inter- and intra-donor variation, suggesting that the observed variation is not technical in nature.

**Table 3:** Coefficient of Variance (% CoV) for whole blood (WB) samples from different donors at the same time point (n = 6), WB samples from the same donors at two different time points (n = 3) and WB samples from the same donor at the same time (n = 4 for tension and n = 3 for compression).

	Tension		Compression	
	Low-strain	High-strain	Low-strain	High-strain
Inter-donor variation (n = 6)	48	49	47	30
Intra-donor variation (n = 3)	N/A	N/A	28	26
Technical repeats (n = 4, n = 3)	16	25	7	13

### Red Blood Cell Content and Clot Contraction

**Figure 5:**
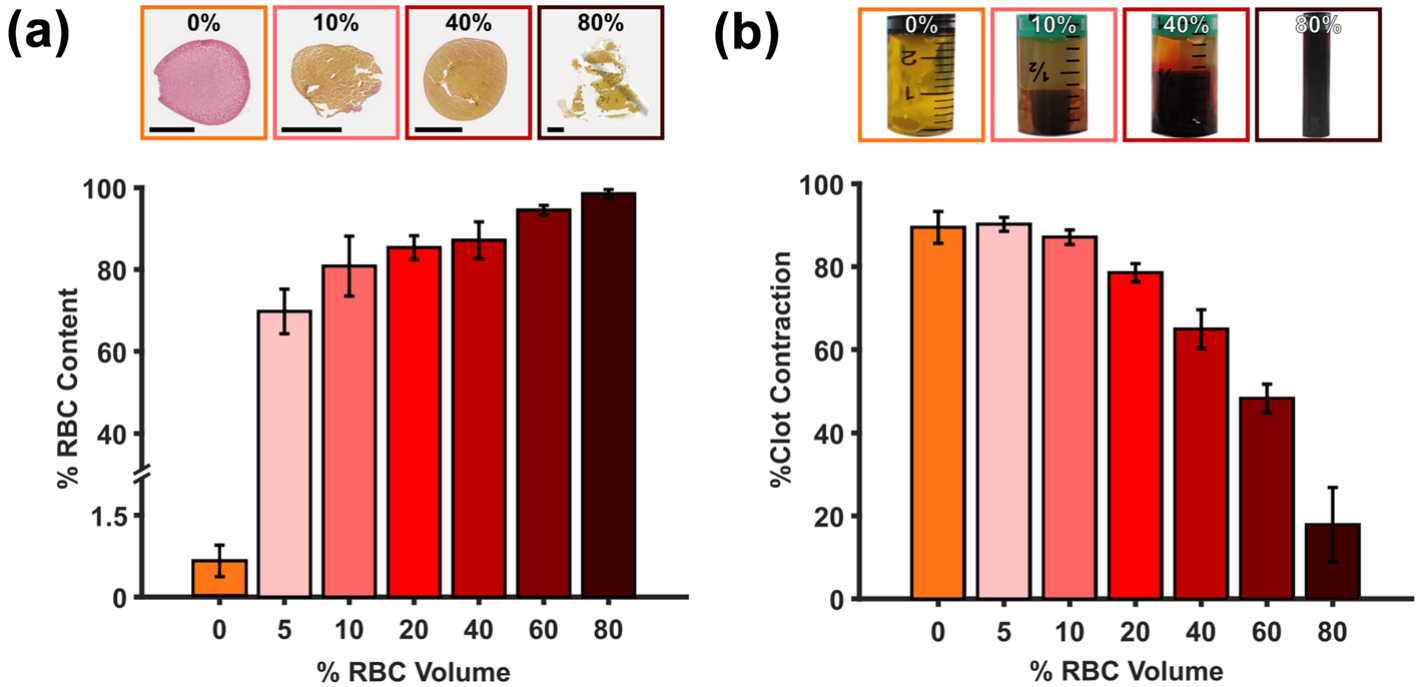
Red blood cell (RBC) content and clot contraction. (a) Variation of RBC content in blood clot analogues and (b) clot contraction by weight per volumetric percentage of RBCs in the blood mixture. All scale bars represent 1 mm.

Figure 5a presents the percentage of RBC content within the clots for each volumetric RBC percentage in the blood mixture. The RBC content in the clots ranged from 0.65% (0% RBC volume blood mixture) to 98.5% (80% RBC volume blood mixture). The RBC volumes in the blood mixture did not produce clot analogues with proportionate RBC contents. It should be noted that even for 5% RBC volume in the blood mixture, the resulting clot contains approximately 70 ± 5% RBCs. Representative stained sections for all compositions are presented in Figure S6. Area fractions for RBCs and other components for each composition are presented in Table S4. There is a strong negative, almost-linear relationship between the volumetric percentage of RBCs in the blood mixture and the degree of clot contraction (*r*_p_ <  − 0.96, *p* < 0.0005 for each donor, Fig. 5b and Table S5).

## Discussion

To the authors’ knowledge, this is the first mechanical characterisation of clot analogues made from the blood of healthy human donors in a range of compositions under both tensile and compressive loading. There is a significant tension–compression asymmetry in clot stress–strain behaviour. At low strains, compressive stiffness is lower than tensile stiffness, while at high strains it is much higher than the tensile stiffness. Tensile high-strain stiffness tends to decrease with increasing RBC volumes while compressive high-strain stiffness increases from 0 to 10% followed by a decrease from 10 to 80% RBC volume in the blood mixture. Finally, our results suggest that up to 50% variation exists in the stiffness of WB clot analogues from different healthy human donors.

Compression-tension asymmetry for blood clots has been suggested based on preliminary data for 5% RBC volume ovine blood clot analogues.^9^ Here we confirm these findings for a wider range of human blood clot analogue RBC volumes. The compressive strain-stiffening response is well established, but data for the tensile response was conflicting.^2, 19^ Strain stiffening under compression likely originates from fibrin fibre densification, the criss-crossing of non-crosslinked fibres^15^ and the resistance to deformation by packed RBCs within the network.^13, 18, 32^ The absence of tensile strain-stiffening can be attributed to the accumulation of network damage as recently described for whole blood clots.^26^ Whole blood clots demonstrate linear behaviour in tension until failure at approximately 70% strain. This is confirmed by the results for WB clots in the current study which also exhibit approximately linear tensile stress–strain profiles and fail in tension between 55 and 80% tensile strain. In practice, this suggests that it becomes increasingly difficult to compress and engage a clot within a stent retriever, however, there is no stiffening mechanism resisting high-strain tensile deformation in the thrombus during device retraction or aspiration. Computational simulations of EVT are expected to play a key role in the future for treatment planning as well as device design and procedural optimisation.^20, 21^ These simulations require material models that accurately represent thrombus properties. Accordingly, our results demonstrate that hyper-viscoelastic damage models^26^ which capture the linear clot response prior to tensile failure and hyperelastic models that capture the strain-stiffening compressive profile will be essential to capture the thrombus multi-axial response to EVT.

Further, our macroscale data suggests that the RBC volume contributes to compressive deformation resistance, but not tensile deformation resistance, most evident in the high-strain stiffness data. We found that high-strain tensile stiffness generally decreased with increasing RBC volumes, similar to another tensile examination of human blood clots where the tensile stiffness of clots at 45% strain also decreased with increasing RBC content.^19^ The formation of polyhedrocytes within contracted clots is a result of applied compressive pressure^30^ on the deformable RBCs,^17^ mediated by platelets pulling on fibrin fibres.^3^ During tensile deformation, it appears that the fibrin network is the primary load-bearing component of the clot. The reduction in stiffness with increasing RBC volumes is likely attributed to the lower proportions of fibrin within the final clots. Although fibrinogen can interact with RBC membrane through integrin-associated proteins,^7^ any clot mechanical consequences of this require examination at the microscale. In compression, we found that the stiffness of blood clots increases from 0 to 10% RBC volume, then decreases with further amounts of RBCs. The process of clot contraction also redistributes the platelets and fibrin to the exterior of the clot.^5^ Upon compressive loading, the polyhedrocytes, which have already undergone a shape change within the contracted clot, and are trapped within the clot matrix by the fibrin on the exterior,^5^ will resist bulk clot compressive deformation. However, at higher RBC volumes we observed less fibrin in the clots. In addition, we observed higher amounts of RBCs on the waterbath stage after the compression test was completed for higher RBC volume clots. Together, these observations suggest that during the compression of higher RBC volume clots, RBCs are squeezed out of the network, and cannot resist compressive deformation as much as the lower RBC volumes. A similar observation of RBC movement within and out of WB clots during compression has been reported previously.^18^ The inflection point at which higher RBC volumes no longer increases the compression stiffness was identified as 10% RBC volume in the current study for human samples. A 20% RBC volume inflection point was previously identified for ovine samples.^13^

In addition to RBC content, we found that clot contraction did not significantly change the stiffness of fibrin clots in tension, but did increase the stiffness in compression, similar to previous findings for ovine clots in compression.^18^ Platelet-driven contraction in PRP clots creates pre-existing internal stresses and a denser fibrin network compared with non-contracted PPP clots. A denser network is more susceptible to higher densification and fibre criss-crossing, explaining the higher compressive stiffness compared with PPP clots. This underscores previous recommendations that thrombus contraction, as well as thrombus composition, should be determined in the clinic to predict EVT response.^2^ A comparison of PPP and PRP response in tension has not been examined previously. Combined mechanical testing with confocal microscopy of labelled fibrin networks^18^ could reveal the origins of this behaviour.

While thrombus composition is linked with thrombus mechanics,^2^ composition alone cannot explain the variation in mechanical properties observed with ex vivo human thrombi acquired from EVT procedures.^1^ Previous data suggests that there is a degree of inter-donor variation in clots prepared in the same manner from the same species.^23^ Here we observed a 30–49% variation in the stiffness of WB clots, in line with the variation of elastic moduli of contracted human whole blood clots 38 ± 15%.^24^ The origin of this mechanical variation is not immediately clear. The biological variation in coagulation activities between individuals likely plays a role.^12^ We did note trends towards significant correlations of platelet cell counts and levels of fibrinogen but with high-strain compressive stiffness only. In terms of intra-donor differences, there are conflicting observations of low^29^ and high^23^ variability in blood clots from the same donor or batch. Here we observed some variation between clots made from the same donor at different times (26–28%), but to a lesser extent than the differences observed between donors (30–49%). This variation in whole blood clot stiffness is expected to be even higher in patient populations.^6, 31^ If whole blood clot analogue mechanical properties are shown to be associated with EVT reperfusion or procedural parameters, there is the potential to introduce a whole blood clot mechanical assay in the AIS diagnosis/treatment workflow to predict behaviour and optimise treatment strategies.

It is known that the volumes of RBCs used in the blood mixture do not produce blood clot analogues with proportionate RBC content,^8, 10, 13^ as underscored by the current study. The differences between the volume of RBCs in the blood mixture and the resulting RBC content of the clot seems to be higher for human^10^ compared with ovine clots.^8, 13^ We speculate that this could be due to the methods of blood component separation. Ovine RBCs are smaller than human RBCs (4–5 *μ*m vs. 6–8 *μ*m),^33^ and could therefore require different centrifuge settings to separate the cells in a comparable nature for blood component volumetric reconstruction. Additionally, the negative relationship between the volumetric percentage of RBCs and the degree of clot contraction with PRP blood mixtures is comparable with ovine studies.^13^ Clot contraction is especially relevant since it alters blood clot mechanics^13^ and is potentially a pathogenic factor in AIS.^31^

There are some limitations that need to be acknowledged. First, a small number of healthy donors were included, which limits the generalisability of the results. Second, clots with 0 and ≥ 70% RBC content do not accurately represent the full range of compositions identified for thrombi retrieved from EVT.^27^ Future studies should prepare blood clot analogues with an intermediate RBC content (0–70%). Third, EVT retrieval induces multi-axial loading at different strain rates on thrombi during retrieval. To simplify the mechanical characterisation of blood clot analogues we conducted uniaxial tensile and unconfined compression experiments, but not shear or viscoelastic experiments. Future studies should perform experiments appropriate for characterising the shear and viscoelastic response of clots. Fourth, clots were prepared under static conditions, therefore have a homogeneous composition. While this is advantageous for mechanical characterisation,^2, 13^ it should be noted that this does not represent all types of *in vivo* thrombi.^27^ Future studies could prepare and characterise blood clots under physiological loading conditions using benchtop flow chambers. Finally, clot mechanics appears to be a function of several variables including RBC and platelet content, fibrinogen concentration and state of contraction.^2^ Future work is needed to decipher the independent and collective contributions of these components to define a metric for predicting clot response to treatment.

In conclusion, pronounced asymmetry was observed in the tension–compression nominal stress–strain response of human blood clot analogues. The effect of RBC volume on the high-strain stiffness of clots differs in tensile versus compressive loading. Finally, there is significant variation present in the stiffness of blood clots prepared from different healthy human donors.

## Supplementary Information

Below is the link to the electronic supplementary material.

Supplementary file1 (PDF 1331 kb)

## Abbreviations

AIS: Acute ischemic stroke
EVT: Endovascular treatment
MSB: Martius scarlet blue
PPP: Platelet-poor plasma
PRP: Platelet-rich plasma
WB: Whole blood
CoV: Coefficient of variance
