# Supplementary material for: Tensile and Compressive Mechanical Behaviour of Human Blood Clot Analogues
Source: Ann Biomed Eng. 2023 Apr 18;51(8):1759–68. doi: 10.1007/s10439-023-03181-6 (PMC10326096; doi:10.1007/s10439-023-03181-6)
Supplement: Supplementary file 1 — Supplementary file1 (PDF 1331 kb) [file 10439_2023_3181_MOESM1_ESM.pdf]

Supplementary Material

METHODS

Uniaxial Tension

Tensile experiments were conducted using a custom-built set-up consisting of a set of clamps, attached to a linear actuator (EACM4-E15-ZAMK, Oriental motor) and a 2.5N load cell (LSB200 Jr. Miniature S-beam load cell, Futek). The tensile moulds were 24mm in length and 10mm wide at the centre of the sample and either 4 or 5mm deep (Fig.1C). However, the final sample length, width and thickness were dependent on the degree of clot contraction. Tensile specimens were removed from the incubator after overnight contraction at 37°C (15.87 ± 0.10 hrs). At the same time, compression specimens were also removed from the water bath and placed at 4°C until the time of testing to prevent any further contraction. Care was taken to remove the tensile specimens from the moulds using the Velcro tabs placed at each end without inadvertently applying stretch to the sample prior to testing (Fig.S2A and B). The samples were then placed in the set of clamps attached to the tensile loading setup To prevent both sample slippage and stress concentrations, the clamps were lined with doubled sided foam tape with P400 grit sandpaper at the bottom and P120 grit sandpaper at the top. A groove was cut into the bottom clamp to create space for the Velcro tabs (Fig.S2C and D). Samples were then inserted into the clamps with the adhesive part of the Velcro facing upwards. A 1.5 cNm torque was applied to each bolt on the clamps (2 bolts per clamp) to fix the sample in position. The water bath was then filled with pre-heated Gibco™ Dulbecco's Modified Eagle Medium (DMEM) or Iscove's Modified Dulbecco's Medium (IMDM), high glucose, HEPES, no phenol red (ThermoFisher Scientific, USA) until the samples were completed submerged. To remove slack from the samples prior to the test, a pre-stretch at a rate of 0.1mm/s was applied until an increase in load was recorded (approximately 0.001N, which is 500 times smaller than the maximum force reading for 60% RBC volume samples and 2500 times smaller than the maximum force reading for PRP sample) (Fig.S2E). The gauge length after applying the pre-stretch was measured to calculate the required velocity to conduct all tests at a rate of 5% strain per second for all samples. Samples were then allowed to equilibrate in the heated physiological solution for at least 5 mins before testing. A scan was conducted along the length of the specimen while the

samples were equilibrating using a Vevo 3100 Imaging System with a MX550S transducer (FUJIFILM VisualSonics, Canada). This scan was later used to determine the specimen's cross-sectional area. Both the pre-conditioning and the stretch cycles were conducted at a strain rate of 5%/s. After the 5 mins equilibration time, samples were pre-conditioning using 10 cycles to 10% strain and then stretched until failure to acquire sample stiffness from both low and high strains. During the test, the time and the corresponding force values were recorded. All tensile mechanical tests were performed within 4.5 hrs of removing the samples from the incubator (4.35 ± 0.22 hrs). In preliminary experiments, it was not possible to perform tensile testing on 80% RBC volume samples. This was due to the fragile nature of the samples. Breakage often occurred while removing the samples from the moulds, or during the handling as depicted in Figure S2F. Therefore, 80% RBC volume samples were not prepared for tensile testing. To examine the reproducibility of the test method, two WB samples were prepared and tested for 4 of the donors (Table S1).

Unconfined Compression

Compression experiments were conducted using a custom-built setup, previously described,[1] with a 450g load cell (LSB200 Jr. Miniature S-beam load cell, Futek) (Fig.1D). Compression syringes were removed from the fridge after 5.82 ± 0.32 hrs of storage. Johnson et al. (2020b) previously found that storage at 4°C in their serum does not affect thrombus properties. The solid portion of the thrombus was removed from the syringe and cut to a height of 2mm using a custom cutting tool. The resulting thrombus has a flat top and bottom surface with a circular cross-sectional shape (Fig.1D). After cutting, the sample was photographed with a ruler while submerged in a physiological solution to perform cross-sectional area measurements using ImageJ. The diameter of all clots was at least 3mm to produce a ratio suitable for compression testing (1.5 times the height). The samples were then transferred to a heated water bath containing DMEM or IMDM at 37°C. The compression plate was lowered to the starting height and the sample was allowed to equilibrate in the heated DMEM for at least 5 mins before testing. Similar to the tensile experiments, samples were pre-conditioned to 10% strain for 10 cycles and then compressed to 80% strain. Both the pre-conditioning and stretch cycles were conducted at 5% strain per second. The time of the experiment and the corresponding force values were recorded during the test. All compression tests were performed within 3 hrs of removing the samples from the

fridge (2.70 ± 0.37 hrs). All 9 compositions underwent compression testing. To examine the reproducibility of the test method, two WB samples were prepared and tested in the compression experiment for 3 of the donors. To conduct a preliminary examination of the intra-donor variation, a second blood draw was taken from donors 1, 3 and 4 at a later time point to make an additional set of samples for compression testing, scanning electron microscopy (SEM) and histology analysis (see Section 4.4 and 4.5). A complete overview of the samples tested is presented in Table S1.

Data Analysis

Cross-sectional measurements were acquired for the samples in the tensile experiments by taking the average of 3 measurements at a proximal, middle and distal location from the length of the ultrasound scan. Cross-sectional measurements were acquired for the samples in the compression experiments by taking 1 measurement of the cross-sectional area from the photographs. All measurements were performed in ImageJ.

For both tensile and compression data, a low-strain stiffness value was acquired by applying a linear fit to the nominal stress-strain data between 0 and 10% strain. For tensile data, a high-strain stiffness value was acquired by applying a linear fit to the final 10% of the stress-strain curve before sample failure (defined as a decrease in the slope of the curve). For compression data, a high-strain stiffness value was acquired by applying a linear fit to the nominal stress-strain data between 70 and 80% strain.[2]

The choice of presenting both low- and high-strain stiffness values originates from the distinct strain-stiffening response of the blood clot analogues under compression, as observed in preliminary experiments. The non-linear nominal compressive stress-strain profiles consist of an initial linear portion at low strains (<20% strain), followed by a non-linear strain-stiffening region and then finally a linear portion again at high strains (>70% strain). The acquisition of both low- and high-strain stiffness values considers the entire response of a blood clot under compression. For tensile data, it is also possible to compute low-strain stiffnesses in the same manner. However, since the tensile samples failed at different strains it was not possible to define the same high-strain region for each sample. Instead, we used the final 10% of the curve from the point of onset of failure (decrease in the curve), similar to a previous approach.[2] Here, our high-strain compressive stiffness value over the 70-80% compression range

is comparable to tangent stiffness at 75% strain, as used in previous studies,[1] since it is in the linear portion of the curve.

FIGURES

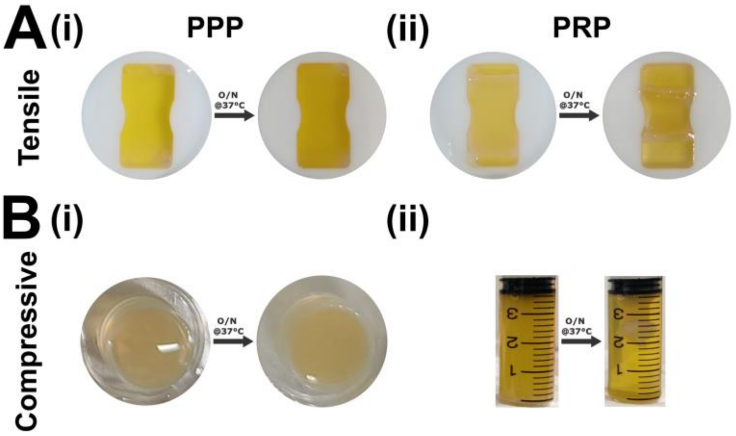

Figure S1: Representative fibrin clot contraction without or with platelets before and after overnight incubation at 37°C. (A) Tensile samples formed in custom-made moulds (i) platelet-poor plasma (PPP) and (ii) platelet-rich plasma (PRP). (B) Compressive (i) PPP samples prepared in Eppendorf lids and (ii) PRP samples prepared in 5mL syringes.

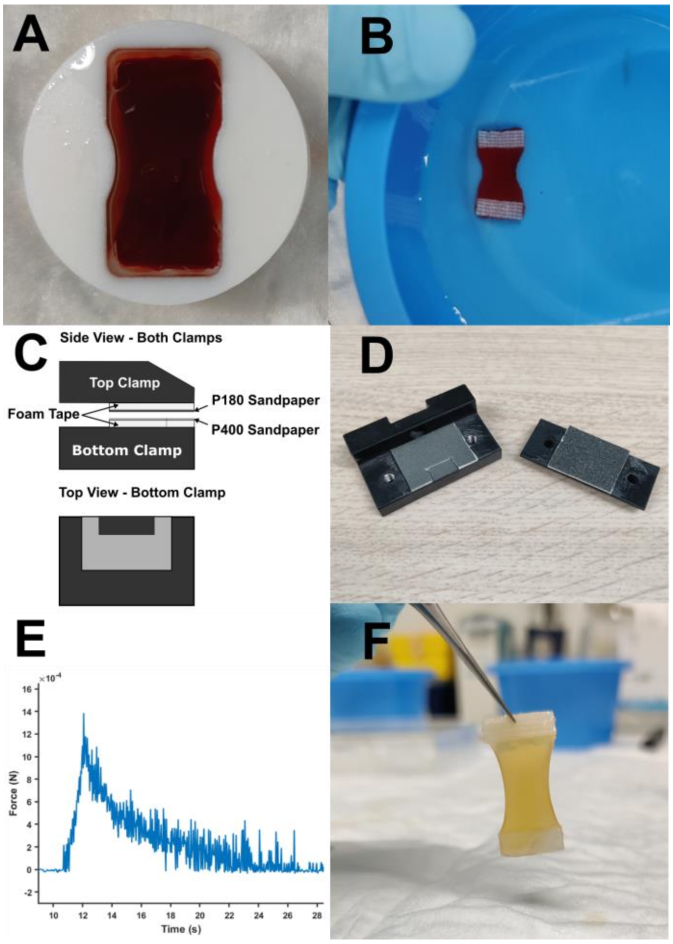

Figure S2: Mechanical characterisation illustrations. (A) Tensile sample in mould. (B) Sample after carefully removing from the mould. (C) Schematic of the tensile clamps. (D) Photo of the clamps. (E) Representative pre-stretch force-time data. (F) Representative PPP sample capable of being removed from the mould without damage.

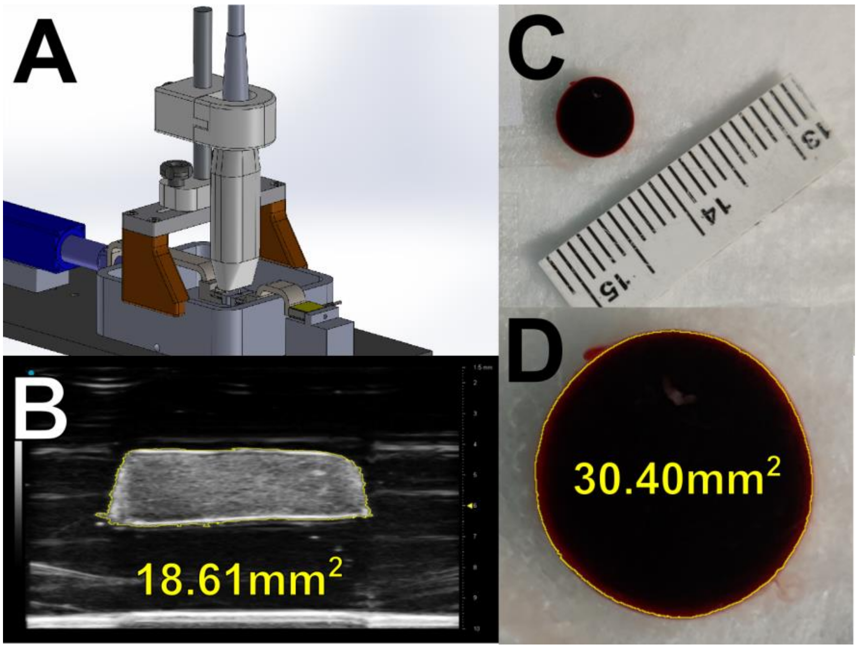

Figure S3: Representative cross-sectional measurements. (A) Ultrasound setup for tensile samples. (B) Representative whole blood (WB) sample cross-section as visualised and measured from the ultrasound images. (C) Photograph of WB compression sample prior to testing. (D) Representative measurement of the cross-sectional area from the photographs.

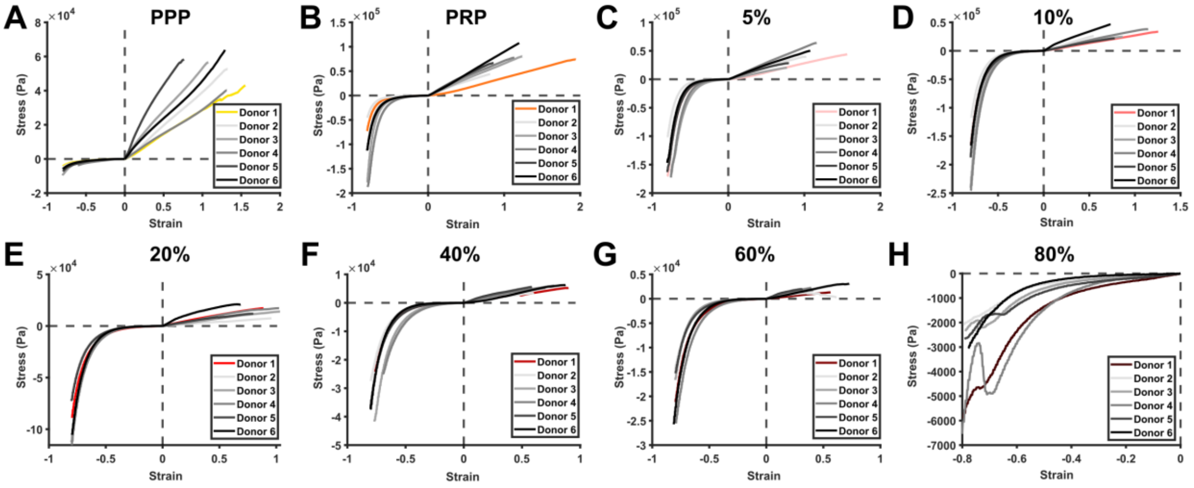

Figure S4: Nominal stress-strain curves for samples in compression (negative strain) and tension (positive strain): (A) platelet-poor plasma (PPP), (B) platelet-rich plasma (PRP), (C) 5%, (D) 10%, (E) 20%, (F) 40%, (G) 60% and (H) 80% red blood cell (RBC) volumes.

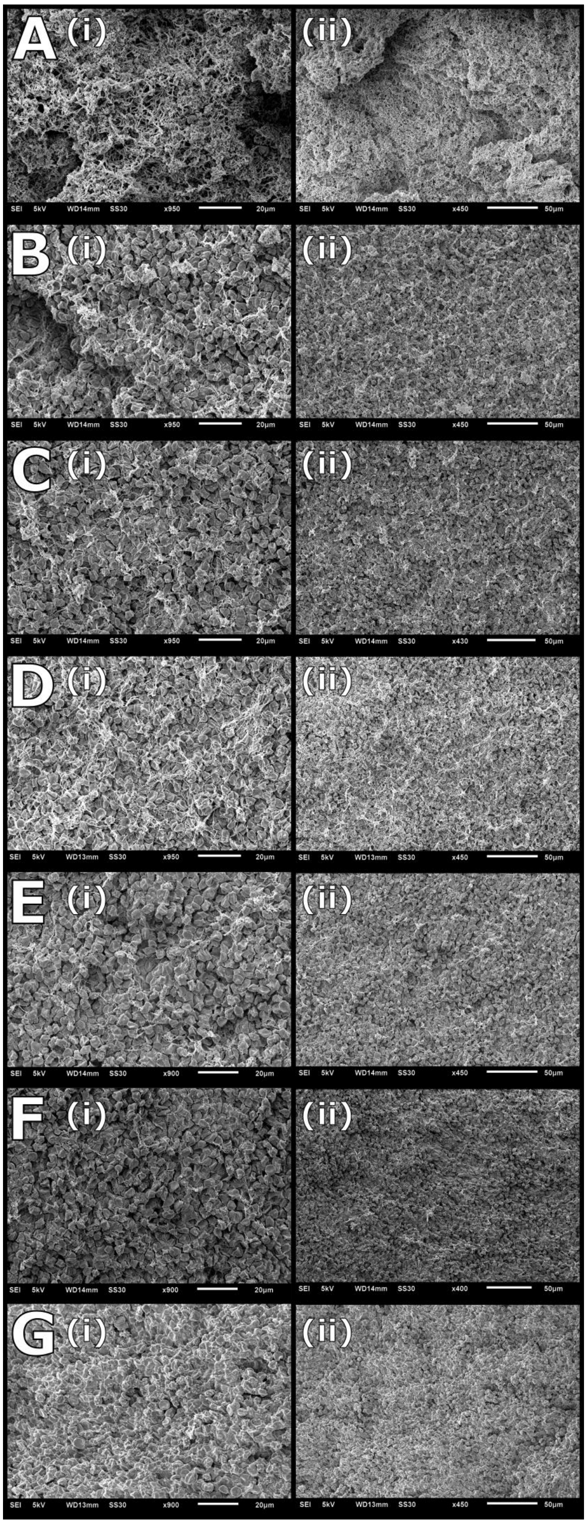

Figure S5: Representative scanning electron microscopy images. (A) Platelet-rich plasma (PRP), (B) 5%, (C) 10%, (D) 20%, (E) 40%, (F) 60% and (G) 80% red blood cell (RBC) volumes with (i) 20μm (x 900-950 magnification) (ii) and 50μm (x 400-450 magnification) scale bars.

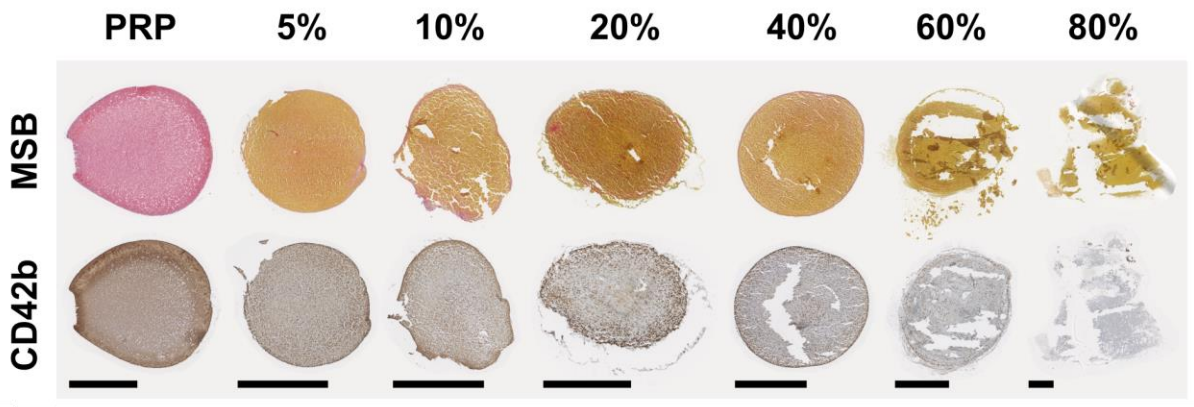

Figure S6: Representative histology (Martius Scarlet Blue [MSB]) and immunohistochemistry (CD42b platelet marker) sections for all compositions (platelet-rich plasma [PRP]). All scale bars represent 1mm.

TABLES

Table S1: The number of samples prepared and tested for each donor at each time point for each type of test. Ex = excluded.

	Donor	PPP	PRP	5%	10%	20%	40%	60%	80%	WB
Tension Testing	1	1	1	1	1	1	Ex	1	0	1 (1 Ex)
	2	1	1	1	1	1	1	1	0	2
	3	1	1	1	1	1	1	1	0	2
	4	1	1	1	1	1	1	1	0	1 (1 Ex)
	5	1	1	1	1	1	1	1	0	2
	6	1	1	1	1	1	1	1	0	2
Compression Testing	1 t1	1	1	1	1	1	1	1	1	2
	2	1	1	1	1	1	1	1	1	1
	3 t1	1	1	Ex	1	1	1	1	1	2
	4 t1	Ex	1	Ex	1	1	Ex	1	1	2
	5	1	1	1	1	1	1	1	1	1
	6	1	1	1	1	1	1	1	1	1
Compression Testing	1 t2	0	0	0	0	0	0	0	0	1
	3 t2	0	0	0	0	0	0	0	0	1
	4 t2	0	0	0	0	0	0	0	0	1

	1 t2	0	1	1	1	1	1	1	1	0
Histology and SEM	3 t2	0	1	1	1	1	1	1	1	0
	4 t2	0	1	1	1	1	1	1	1	0

Table S2: Coefficient of variance (% CoV) for other measured parameters for each donor.

Donor	Platelets (x 10^3 cells/µL)	Hematocrit (Fraction)	White Blood Cells (x 10^3 cells/µL)	Fibrinogen (g/L)	Whole Blood Clot Contraction (%)
1	262	0.40	6.00	2.60	69.66
2	185	0.44	6.47	2.30	55.91
3	192	0.39	7.10	2.70	66.33
4	244	0.42	5.77	3.30	61.70
5	160	0.52	4.77	2.80	63.73
6	242	0.45	7.10	4.20	55.43
CoV (%)	19	11	14	23	9

Table S3: Pearson's correlation coefficients for donor demographics and blood composition with mechanical stiffness.

	Tensile				Compressive			
	Low-strain		High-strain		Low-strain		High-strain	
	r_p	*P*-value	r_p	*P*-value	r_p	*P*-value	r_p	*P*-value
Age	0.02	0.97	0.63	0.18	0.31	0.55	**0.79**	**0.06**
Hematocrit	0.08	0.88	-0.09	0.87	0.05	0.92	-0.31	0.55
Platelets	-0.29	0.58	0.41	0.43	0.28	0.60	**0.74**	**0.09**
White Blood Cells	-0.54	0.27	-0.28	0.59	-0.49	0.33	0.10	0.85
Fibrinogen	-0.22	0.68	0.30	0.56	0.11	0.84	0.71	0.12
Contraction	0.45	0.37	-0.11	0.84	**0.78**	**0.07**	0.18	0.74

Table S4: Quantitative compositions for each type of blood clot analogue as determined histologically from Martius Scarlet Blue (MSB) and CD42b stained sections.

	MSB %RBCs	CD42b %Platelets	CD42b %WBCs
0%	0.65 ± 0.30	89.83 ± 3.40	2.01 ± 0.62
5%	69.74 ± 5.46	38.75 ± 16.90	1.74 ± 0.90
10%	80.82 ± 7.33	42.43 ± 5.42	3.22 ± 1.57
20%	85.34 ± 2.93	33.95 ± 1.04	5.63 ± 2.32
40%	87.14 ± 4.52	25.42 ± 2.60	3.37 ± 3.47
60%	94.52 ± 1.17	13.62 ± 2.62	1.64 ± 0.06
80%	98.50 ± 1.06	1.43 ± 0.48	0.46 ± 0.35

Table S5: Pearson's correlation coefficients per donor between the percentage volume of red blood cells in the reconstructed blood and the percent clot contraction.

Donor	1	2	3	4	5	6
r_p	-0.962	-0.984	-0.968	-0.998	-0.977	-0.981
P-value	0.0005	0.0001	0.0004	0.0000	0.0002	0.0001

SUPPLEMENTAL REFERENCES

1. Boodt, N., P. Snouckaert van Schauburg, H. Hund, B. Fereidoonnezhad, P. McGarry, A. Akyildiz, A. van Es, S. De Meyer, D. Dippel, H. Lingsma, H. van Beusekom, A. van der Lugt, and F. Gijsen. Mechanical Characterization of Thrombi Retrieved with Endovascular Thrombectomy in Patients with Acute Ischemic Stroke. Stroke , 2021.doi:10.1161/STROKEAHA.120.033527

2. Johnson, S., J. Chueh, M. J. Gounis, R. McCarthy, J. P. McGarry, P. E. McHugh, and M. Gilvarry. Mechanical behavior of in vitro blood clots and the implications for acute ischemic stroke treatment. J. Neurointerv. Surg. 1–6, 2019.doi:10.1136/neurintsurg-2019-015489

3. Johnson, S., R. McCarthy, M. Gilvarry, P. E. McHugh, and J. P. McGarry. Investigating the Mechanical Behavior of Clot Analogues Through Experimental and Computational Analysis. Ann. Biomed. Eng. , 2020.doi:10.1007/s10439-020-02570-5
